# Avian Influenza Risk Perception, Europe and Asia

**DOI:** 10.3201/eid1302.060303

**Published:** 2007-02

**Authors:** Onno de Zwart, Irene K. Veldhuijzen, Gillian Elam, Arja R. Aro, Thomas Abraham, George D. Bishop, Jan Hendrik Richardus, Johannes Brug

**Affiliations:** * Municipal Public Health Service, Rotterdam, the Netherlands; † Health Protection Agency—Centre for Infections, London, United Kingdom; ‡ University of Southern Denmark, Esbjerg, Denmark; § University of Hong Kong, Hong Kong Special Administrative Region, People’s Republic of China; ¶ National University of Singapore, Singapore; # Erasmus University Medical Center Rotterdam, Rotterdam, the Netherlands

**Keywords:** Avian influenza, influenza pandemic, risk perception, risk communication, behavior change, dispatch

## Abstract

During autumn 2005, we conducted 3,436 interviews in European and Asian countries. We found risk perceptions of avian influenza to be at an intermediate level and beliefs of efficacy to be slightly lower. Risk perceptions were higher in Asia than Europe; efficacy beliefs were lower in Europe than Asia.

The possibility of an influenza pandemic presents a major public health challenge. Since 2003, outbreaks of avian influenza (AI) have occurred in Asian, European, and African countries. As of August 21, 2006, the total number of cases was 240 and the number of deaths was 141 (*1*). A crossover of current human influenza virus with the avian H5N1 virus could result in a virus capable of human-to-human transmission and the start of a new pandemic.

Despite extensive media attention for avian influenza, knowledge about risk perception of AI is scarce. We therefore explored the conditions for effective nonmedical interventions. If an influenza pandemic occurs, public health authorities will be dependent on the willingness and ability of the public to adhere to recommendations regarding personal hygiene, vaccination and prophylaxis, quarantine, travel restrictions, or closing of public buildings (*2*,*3*). Adherence, however, cannot be assumed. Evaluation of the outbreak of H7N7 AI in the Netherlands in 2003 showed that adherence to antiviral therapy and behavioral measures, such as wearing face masks and goggles, was low (*4*).

Our ability to promote health-protective behavioral change depends on our knowledge of determinants of such behavior (*5*). The protection motivation theory posits that health-protective actions are influenced by risk perceptions (*6*–*8*). Risk perceptions are defined by the perceived seriousness of a health threat and perceived personal vulnerability. However, the protection motivation theory explicitly states that higher risk perceptions will only predict protective behavior when people believe that effective protective actions are available (response efficacy) and that they have the ability to engage in such protective actions (self-efficacy).

## The Study

We investigated risk perceptions and efficacy beliefs related to AI of a random sample of persons in 8 areas. Random digital dialing was used to select the samples, and data were collected by using computer-assisted telephone interviewing. Interviews were conducted from September 20 through November 22, 2005, in 5 European countries (Denmark, the Netherlands, United Kingdom, Spain, and Poland) and 3 East Asian areas (Singapore; Guangdong Province, People’s Republic of China; and Hong Kong, Special Administrative Region, People’s Republic of China). At the time the telephone survey was conducted, on October 14, 2005, the media announced the introduction of AI in Europe. We therefore ensured that at least 90 interviews were conducted in each country after October 18, 2005. The questionnaire focused on risk perception of AI and other infectious diseases, precautionary behavior, and use of information sources; it was based on our earlier study of risk perception of severe acute respiratory syndrome (SARS) (*9*). Respondents first received a brief explanation of AI.

In line with the protection motivation theory (*8*), a measure of risk perception was constructed by multiplication of seriousness (scale 1–10) and vulnerability (scale 1–5). To make the scores comparable, the seriousness score was first divided by 2. To normalize the skewed distribution of the new variable, a square-root transformation was performed, which resulted in a measure of risk perception on a scale from 1 (low) to 5 (high).

A total of 3,436 respondents were interviewed; participation rates varied from 12.9% in Asia to 81.1% in Poland. Most respondents were female (Table 1). European respondents were significantly older than Asian respondents (mean age 47 and 39 years, respectively, range 18–75 years, t = 16,2; degrees of freedom [df] = 3,351; p<0.001). Overall, 45% of respondents thought they were likely or very likely to become infected with AI if an outbreak occurred in their country. This perception varied from 32% in Denmark and Singapore to 61% in Poland and Spain. Risk perception scores varied significantly across countries; the highest mean score was in Poland and the lowest was in Denmark (Table 2). Higher scores were observed in Europe than in Asia (t = 5.2; df = 3,250; p<0.001), and differences between individual countries within Europe were significant. Multivariate analysis showed that country, sex, and age group remained independent significant factors and showed a significant interaction between country and sex and between country and age group (Figure). In all countries, except Singapore, risk perception was higher among women than men, but this difference was smaller in Asian than in European countries. The effects of age also varied by country; mean risk perception levels were higher in older age groups in Europe but not in Asia.

**Table 1:** Distribution of general characteristics of the study population, by country or region, September 20–November 22, 2005*

	No. (%)										
Characteristic	DNK	POL	NLD	UK	ESP	CHN	HKG	SGP	Europe	Asia	Total
Total	463 (14)	488 (14)	400 (12)	401 (12)	425 (12)	404 (12)	396 (12)	426 (13)	2,177 (64)	1,226 (36)	3,403 (100)
Sex											
Male	40	39	42	41	41	47	44	43	40	45	42
Female	60	61	58	59	59	53	56	57	60	55	58
Age group, y											
18–30	13	18	10	13	17	43	27	35	14	35	22
31–44	31	31	31	35	34	34	35	31	32	33	33
45–60	36	32	37	31	32	19	27	20	33	22	29
61–75	20	19	24	21	17	4	12	14	20	10	16
Area											
City	26	21	9	20	45	86	90	81	24	86	46
Town	38	25	37	45	42	9	4	16	37	10	27
Village/countryside	37	54	55	36	13	4	6	2	39	4	26
Education											
Primary or less	17	8	5	2	22	4	13	3	11	7	9
Low	31	22	28	20	9	19	20	11	22	16	20
Intermediate	38	43	35	35	31	35	32	38	37	35	36
High	13	28	32	43	38	42	35	48	30	42	34

*DNK, Denmark; POL, Poland; NLD, the Netherlands; UK, United Kingdom; ESP, Spain; CHN, China; HKG, Hong Kong; SGP, Singapore.

**Table 2:** Perceived risk perception and efficacy beliefs with regard to a potential influenza outbreak, September 20–November 22, 2005*

	Mean score scale 1–10 (95% CI)				
Country or region	Seriousness,	Vulnerability	Risk perception†	Response efficacy	Self-efficacy
DNK	6.08 (5.83–6.33)	2.82 (2.71–2.92)	2.73 (2.65–2.81)	2.32 (2.23–2.41)	2.15 (2.06–2.24)
POL	7.49 (7.29–7.70)	3.43 (3.31–3.54)	3.48 (3.39–3.57)	2.55 (2.46–2.64)	2.06 (1.96–2.16)
NLD	7.67 (7.48–7.87)	3.17 (3.07–3.27)	3.40 (3.32–3.48)	2.25 (2.14–2.35)	1.74 (1.66–1.83)
UK	7.38 (7.16–7.61)	2.93 (2.81–3.05)	3.17 (3.07–3.26)	2.41 (2.32–2.51)	2.03 (1.93–2.12)
ESP	6.76 (6.53–6.99)	3.43 (3.32–3.53)	3.29 (3.20–3.37)	2.75 (2.65–2.85)	2.26 (2.15–2.36)
CHN	6.58 (6.33–6.82)	2.88 (2.76–2.99)	2.94 (2.85–3.04)	2.99 (2.92–3.06)	2.90 (2.82–2.99)
HKG	7.02 (6.81–7.23)	3.33 (3.23–3.42)	3.33 (3.25–3.40)	2.95 (2.87–3.03)	2.64 (2.55–2.73)
SGP	6.63 (6.35–6.91)	2.70 (2.57–2.83)	2.82 (2.71–2.93)	2.81 (2.71–2.91)	2.70 (2.61–2.80)
Europe‡	7.06 (6.96–7.16)	3.16 (3.11–3.21)	3.21 (3.17–3.25)	2.46 (2.41–2.50)	2.05 (2.01–2.10)
Asia	6.74 (6.60–6.88)	2.97 (2.90–3.03)	3.03 (2.97–3.08)	2.92 (2.87–2.96)	2.75 (2.69–2.80)
Total	6.95 (6.86–7.03)	3.09 (3.05–3.13)	3.14 (3.11–3.17)	2.63 (2.59–2.66)	2.31 (2.27–2.34)

*CI, confidence interval; DNK, Denmark; POL, Poland; NLD, the Netherlands; UK, United Kingdom; ESP, Spain; CHN, China; HKG, Hong Kong; SGP, Singapore. †Square root of the multiplication of seriousness divided by 2 and vulnerability. ‡Differences in mean scores between Europe and Asia are significant for all measures (p<0.001).

**Figure:**
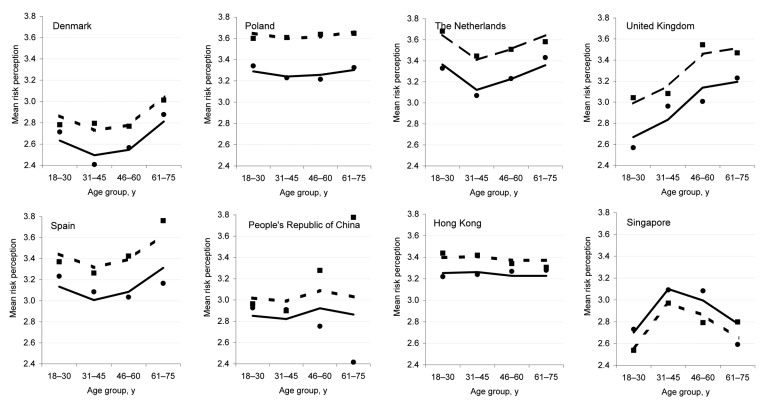
Mean risk perception by country or region, sex, and age group. Lines, predicted means; dots, observed means; solid line, male; dashed line, female.

Response efficacy and self-efficacy also varied across countries; levels were highest in China and lowest in the Netherlands (Table 2). Mean response efficacy and self-efficacy were significantly higher in Asia than in Europe (response efficacy t = −14; df = 2,868; p<0.001; self-efficacy t = −20; df = 2,701; p<0.001). Response and self-efficacy were inversely associated with risk perception (p = 0.013 and p<0.001, respectively).

Multivariate analysis also showed that country, but not sex or age, was significantly associated with response efficacy. Country, sex, and age group were all significantly associated with self-efficacy. Self-efficacy levels were lower for women compared with men and for the youngest age group compared with older respondents. Risk perception and efficacy levels before and after the introduction of avian influenza in Europe did not differ significantly.

## Conclusions

Our study showed that risk perceptions for AI appear to be at an intermediate level and that efficacy beliefs are slightly lower. Both differ according to country or region. No evidence was found that the introduction of AI in Europe in October 2005 influenced perceptions of risk or efficacy.

Fielding et al. have reported on risk perception of AI in Hong Kong with a focus on live chicken sales (*10*). Although our results are difficult to compare with theirs, our study appears to indicate a higher feeling of vulnerability, with 41.8% of Hong Kong respondents thinking it likely or very likely that they would become infected with influenza during an outbreak. Takeuchi’s interviews on food safety practices of consumers in Thailand found high levels of knowledge of AI but lower levels of risk perception and behavior change (*11*). If we compare our results with those from several studies on perception of risk for SARS, we find that perception of risk for SARS in some of the Asian countries was relatively low compared with that in the United States (*12*). In the Netherlands, however, perception of risk for SARS was low, whereas our present study indicates that it is high for influenza (*9*).

The lower level of risk perception for AI in Asia may be related to the proximity to the current outbreak and the experience with the SARS epidemic. These experiences may have resulted in the notion that new epidemics of infectious diseases can be controlled. Also, despite the fact that the first cases of H5N1 influenza among humans in Asia were reported in 2003, a larger outbreak did not ensue. Accordingly, risk perception research has shown that the public may be more optimistic when familiar risks are perceived to be largely under volitional control (*13*,*14*).

Our study has several implications for public health policy and research. Although in all countries an influenza pandemic is perceived as a real risk, the level of self-efficacy appears to be rather low. When developing preparedness plans for an influenza pandemic, specific attention should therefore be paid to risk communication and how perceived self-efficacy can be increased; otherwise, adherence to preventive measures may be low.
